# Traditional Uses, Chemical Constituents, and Biological Activities of *Bixa orellana* L.: A Review

**DOI:** 10.1155/2014/857292

**Published:** 2014-06-23

**Authors:** Daniela de Araújo Vilar, Marina Suênia de Araujo Vilar, Túlio Flávio Accioly de Lima e Moura, Fernanda Nervo Raffin, Márcia Rosa de Oliveira, Camilo Flamarion de Oliveira Franco, Petrônio Filgueiras de Athayde-Filho, Margareth de Fátima Formiga Melo Diniz, José Maria Barbosa-Filho

**Affiliations:** ^1^Laboratory of Pharmaceutical Technology, Federal University of Paraíba, 58051-900 João Pessoa, PB, Brazil; ^2^Department of Pharmacy, Federal University of Rio Grande do Norte, 59010-180 Natal, RN, Brazil; ^3^Department of Molecular Biology, Federal University of Paraíba, 58051-900 João Pessoa, PB, Brazil; ^4^State Company of Agricultural Research of Paraíba, Rua Eurípedes Tavares 210, Tambiá, 58013-290 João Pessoa, PB, Brazil; ^5^Department of Chemistry, Federal University of Paraíba, 58051-900 João Pessoa, PB, Brazil

## Abstract

*Bixa orellana* L., popularly known as “urucum,” has been used by indigenous communities in Brazil and other tropical countries for several biological applications, which indicates its potential use as an active ingredient in pharmaceutical products. The aim of this work was to report the main evidence found in the literature, concerning the ethnopharmacology, the biological activity, and the phytochemistry studies related to *Bixa orellana* L. Therefore, this work comprises a systematic review about the use of *Bixa orellana* in the American continent and analysis of the data collected. This study shows the well-characterized pharmacological actions that may be considered relevant for the future development of an innovative therapeutic agent.

## 1. Introduction

The use of natural compounds of mineral, animal, or plant origin in food products, cosmetics, and drugs began long ago. There are written records of ancient Egyptian and Chinese civilizations that have made use of these products. Nowadays, there has been a return to the search for products called “natural,” which in fact never ceased to exist. The analysis of the composition of many drugs shows that almost 50% of those in clinical use are derived from natural compounds. Furthermore, not only plants but also plant byproducts are widely used as preservatives and flavoring and coloring agents in various food and cosmetic preparations [1].

*Bixa orellana* is a plant native to Brazil but grows in other regions of South and Central America. It is grown in tropical countries such as Peru, Mexico, Ecuador, Indonesia, India, Kenya, and East Africa [2].

The seeds of this plant produce one of the dyes most frequently used worldwide, not only in food products but also in the textile, paint, and cosmetic industries. Its use has been stimulated due to the ban on the use of synthetic dyes in food and cosmetics, where it is one of the few accepted by the World Health Organization (WHO), since, in addition to being nontoxic, it does not seem to change the food value [3]. Another interesting fact is that 70% of all natural coloring agents consumed worldwide are derived from annatto [4].

Annatto first spread in the form of food coloring, also known as paprika, a condiment widely used in cooking to enhance the color of food. Today, however, its use has spread into many segments of industrial production. Thus, it is now applied on the skin—in the form of makeup and sunscreen—and there is research proving that its use brings health benefits, which makes producers thankful for cultivating it [5, 6]. Therefore, in the continuation of our research on bioactive molecules from various species of different plant families [7–22], we offer this compilation of the traditional uses, chemical constituents, and biological activities of* Bixa orellana*.

The aim of this review is to highlight the biological and phytochemical studies that have been published about* Bixa orellana* in South and Central America and try to correlate these studies with the popular uses of this plant in those regions, as well as to evaluate whether its chemical composition can support the reported biomedical properties related to* Bixa orellana*.

## 2. Materials and Methods

In this work, the biological activities and compounds isolated from* Bixa orellana* were searched using the database of the Web of Science, Scielo, and the University of Illinois in Chicago NAPRALERT (acronym for “NAturalPRoducts ALERT”). The data were updated in April 2014, using “*Bixa orellana*, chemical, and bixin” as keywords for this review. The references found in the survey were later consulted for details about the models or mechanisms of bioassays used to test the extracts of* Bixa orellana*.

## 3. Botanical

The annatto tree belongs to the family Bixaceae and the genus* Bixa*. Despite the existence of several species, the most common in our country is* Bixa orellana* L., named after Francisco Orellana, who was the first European to navigate the Amazon [23].

According to Revilla [58],* B. orellana* is a small tree or shrub measuring from 3 to 5 meters in height, sometimes reaching a height of 10 meters. The trunk is short, measuring 20–30 cm in diameter, with dark gray bark with lenticels in vertical rows. The leaves are alternate, 10 to 20 cm long and 5 to 10 cm wide, sharp, green on both sides, and with extended petioles.

According to Oliveira et al. [59], seeds measure 0.3–0.5 cm in length and 0.2-0.3 cm in diameter, and their shape varies from pyramidal to almost conical. The number of seeds per capsule varies according to the author: Alonso [60] found that each bivalvar capsule may contain from 30 to 60 seeds, on average.

The seeds are considered the plant part of commercial importance, since the pericarp (layer that surrounds the seeds) contains the pigments that have wide industrial application. About 80% of this pigment is the carotenoid known as bixin, which has the dye property and can be extracted with vegetable oils or chemical bases. Depending on the cultivar and climatic conditions of the region, the bixin content can vary from 1 to 6% in the seed aril. The remainder is composed of other dyes and inert substances of minor importance [61].

## 4. Use in Traditional Medicine

Annatto is a native plant from South America, more specifically of the Amazon region. The popular name “urucum” comes from the Tupi word “ru-ku,” which means “red.” In Brazil, this plant is commonly known as urucum, urucu, açafrão, açafroa, and açafroeira-da-terra. It is known by other popular names in other countries: atolé, achiote, and bija (Peru and Cuba); axiote (Mexico); achiote, achote, anatto, bija, and santo-domingo (Puerto Rico); bixa (Guyana); analto (Honduras); guajachote (El Salvador); onotto and onotillo (Venezuela); achiote and urucu (Bolivia); urucu (Argentina); roucou (Trinidad); roucou and koessewee (Suriname); and annatto (United States). The wide dissemination of its use in those regions is related to the growing demand for natural dyes by many pharmaceutical, cosmetic, textile, and especially food industries [57].

According to Côrrea [27], seeds urucum supplies seeds that have been used as a condiment as well as laxative, cardiotonic, hypotensive, expectorant, and antibiotic. In addition, it has anti-inflammatory activity for bruises and wounds and has been used for the treatment of bronchitis and for wound healing purposes. Oil is also obtained from this plant. The infusion of the leaves has been shown to be effective against bronchitis, sore throat, and eye inflammation. The pulp, which includes the seed, is used for soft drinks and febrifuge. Moreover, it can provide valuable dyeing materials such as yellow (orellin) and red (bixin) substances, with the latter constituting a crystallized active ingredient.

In the food industry, it is used to color butter, margarine, mayonnaise, sauces, mustard, sausage, soup, juice, ice cream, bakery products, macaroni, and cheese, where it is commonly called “do reino” (of the kingdom), coming from Holland. It is also widely used in the printing industry and dye manufacturing. Many Aborigines use annatto for dyeing, where the dye is naturally obtained as a mixture and used to color ceramics and other vases for domestic use. In addition, most endogenous people use this dye on their skin to beautify themselves during religious rituals and mainly to protect themselves from ultraviolet radiation and from mosquitoes that infest forests [49]. The bast provides fibers for rough cordage, and the powder resulting from grinding the seeds has been used as an aphrodisiac. Finally, the infusion of cold buds serves to wash inflamed eyes, whereas the decoction of the leaves has been used for antiemetic therapy during pregnancy [27] (Table 1).

Thus, despite the different culture and traditions among the countries in South and Central America, several of the popular uses of* Bixa orellana* are the same, for example, antipyretic, aphrodisiac, antidiarrheal, antidiabetic, and insect repellent.

## 5. Chemical Compounds

Bixin, a red-colored carotenoid, is the pigment present in high concentration in the annatto seed aril. It is the main substance responsible for the dyeing characteristics of seeds, where its concentration can be as high as 5.0%. However, different seeds may have levels less than 2.0%, and because their commercial value is based on the bixin percentage, levels higher than 2.5% are usually required for export [61].

Bixin was isolated for the first time from the seeds of* Bixa orellana* in 1875 and in 1961 its complete chemical structure and stereochemistry were determined by ^1^H and ^13^C-NMR. Bixin belongs to the small class of natural apocarotenoids, whose formation occurs by the oxidative degradation of C40 carotenoids (Table 2).

**Figure 1:**
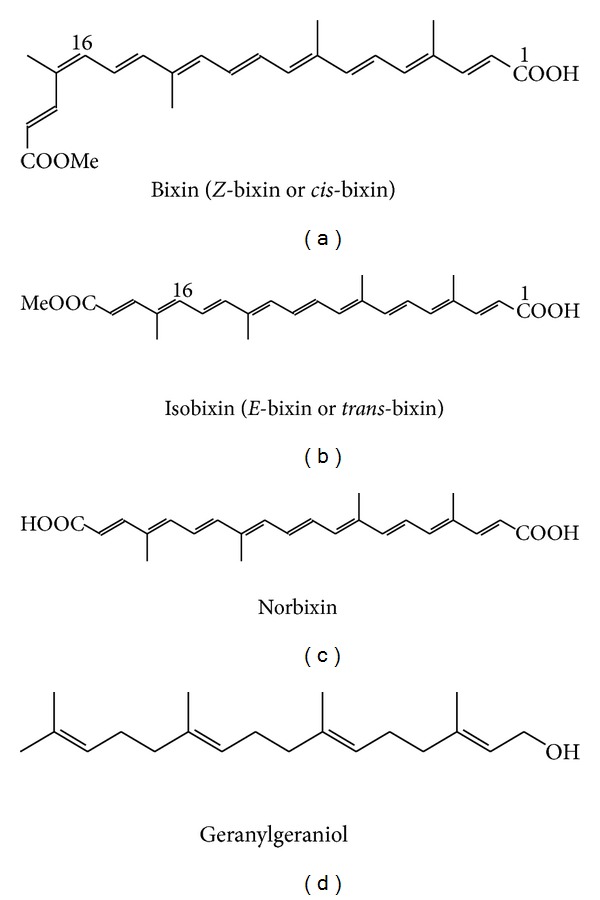
Chemical structure of some pigments of annatto.

Bixin consists of a chain of 25 carbons and has the molecular formula C_25_H_30_O_4_ (MW = 394.51). It has a carboxylic acid and methyl ester group at the ends of the chain. Bixin occurs in nature as 16-*Z* (*cis*), but during the extraction process it isomerizes resulting in the 16-*E* form (*trans*), which is called isobixin (Figure 1).

Many other carotenoids (C_19_, C_22_, C_24_, C_25_, C_30_, and C_32_) occur in* Bixa orellana* but constitute a minor percentage of the pigments. The major oily constituent of annatto seeds is geranylgeraniol, representing 1% of dry seeds. Norbixin (Figure 1) is a demethylated derivative of bixin and although it is a naturally occurring compound, it is almost always referred to as a saponification product of bixin. This is the form used for commercial purposes [62].

Currently, more than two dozen substances have been isolated from the seeds of* Bixa orellana*. Besides bixin and norbixin, other compounds such as isobixin, beta-carotene, cryptoxanthin, lutein, zeaxanthin, orellin, bixein, bixol, crocetin, ishwarane, ellagic acid, salicylic acid, threonine, tomentosic acid, tryptophan, and phenylalanine have been found in the seeds of annatto. In addition, the following compounds, in their respective concentrations, are found in these seeds: 40 to 45% cellulose, 3.5 to 5.5% sugars, 0.3 to 0.9% essential oils, 3% fixed oils, 1.0 to 4.5% pigments, and 13 to 16 % proteins and* alpha* and* beta*-carotene, as well as tannins and saponins [63, 64].

Mercadante et al. [50, 65] isolated eight apocarotenoids from annatto seeds: methyl (7Z, 9Z, 9′Z)-apo-6′-lycopenoate, methyl (9Z)-apo-8′-lycopenoate, methyl 1(all-E)-apo-8′-lycopenoate, methyl (all-E)-8-apo-beta-carotene-8′-oate, methyl (all-E)-apo-6′-lycopenoate, 6-geranylgeranyl-8′- methyl-6,8′diapocaroten-6-8′dioate, 6′-geranylgeranyl-6′-methyl-(9Z)-6,6′-diapocaroten-6-6′-dioate, and 6-geranylgeranyl-6′-methyl-6-6′-diapocaroten-6-6′-dioate.

More than 100 volatile compounds have been detected in aqueous and organic extracts, where 50 of these have already been identified (e.g., bornyl acetate, *∝*-caryophyllene, copaene, *∝*-cubebene, (+)-cyclosativene, geranyl phenylacetate, 1-heptanetiol, 3-methylpyridine, 4-methylpyridine *γ*-elemene, *β*-humulene, isoledene, *β*-pinene, seline-6-en-4-ol, *δ*-selinene, (−)-spathulenol, and (+)-ylangene) [66].

Because annatto is a rich source of carotenoids it is of great commercial importance. In fact, the therapeutic properties of annatto (e.g., antioxidant and hypoglycemic) have been attributed to its high levels of carotenoids [67–69]. Table 2 lists some of these compounds.

The pigments in annatto seeds can be extracted by mechanical processes through grinding the seeds and by physical-chemical methods using solvents or enzymes [46]. The solvent extraction can be performed using three basic methods: alkaline extraction (NaOH or KOH solutions), which results in the conversion of bixin to norbixin; extraction with oil (soybean, corn); and extraction using organic solvents (hexane, chloroform, ethanol, acetone, or propylene glycol), which results in the purest form of pigments.

Barbosa-Filho et al. [49] studied the seeds of four types of annatto cultivated in Paraíba State, Brazil, namely, “cascaverde” (“green peel”), “cascavermelha” (“red bark”), “bico de calango” (“lizard beak”), and “grãopreto”(“black grain”), with respect to their oil (material extracted with hexane) and solid (material extracted with chloroform) contents, and also pure bixin, which was obtained by successive recrystallization from the chloroform fraction. Pure bixin appears as red-purple crystals with a melting point of 196–198°C. The different concentrations found for the oil fraction, chloroform extract, and bixin are as follows: red bark 5.8%, lizard beak 5.1%, green peel 4.9%, and black grain 4.6%. Red bark shows the highest yield for both solvent fractions, and the bixin amount is around 1%. This species has been reported as the most used in folk medicine. On the other hand, black grain shows negligible amounts of bixin.

## 6. Biological Activity

Table 3 shows data found in 38 studies performed with annatto in 15 different countries in the American countries. To obtain the extracts and fractions tested, several plant parts were used, such as leaf, root, seed, shoot, and even the whole plant. The data surveyed were classified according to the pharmacological activity tested.

Among the twenty-one activities tested, those with the largest number of studies performed were antifungal activity (12), antibacterial activity (12), antimalarial activity (6), and mutagenic activity (3). Cytotoxic activity and toxicity have been little studied, with three and two studies, respectively. Pharmacological activities have been evaluated in animal models (22 preclinical studies), human models (1 clinical study), cell cultures (2 studies), and* in vitro *tests (32 studies).

Antifungal activity has been investigated in one country in Central America (Guatemala) and in two countries in South America (Ecuador and Argentina) using eleven different fungal strains [70, 82, 84].

Freixa et al. [82] conducted a study in Ecuador to assess the antifungal activity of extracts from the dried leaves of the annatto tree in response to 7 fungi species, obtaining satisfactory antifungal activity against* Trichophyton mentagrophytes* trains. In Guatemala, three different strains were used to evaluate antifungal activity, with no satisfactory activity being observed [84].

The extracts of annatto leaves have been evaluated for antibacterial activity against 8 different bacterial strains (*Bacillus subtilis, Escherichia coli, Micrococcus luteus, Pseudomonas aeruginosa, Staphylococcus aureus, Salmonella typhi, Shigella dysenteriae,* and* Staphylococcus epidermidis*), showing no activity.

Antimalarial activity has been determined against* Plasmodium gallinaceum*,* Plasmodium lophurae,* and* Plasmodium berghei*. Although the studies conducted previously in the United States did not show significant results [92], Valdés et al. (2011) reported a moderate activity of the seed extracts of* Bixa orellana* against* Plasmodium berghei* and* falciparum*.

## 7. Mutagenic and Cytotoxic Activities

No significant effect was observed when extracts of annatto seeds were tested for mutagenic activity in studies performed in the United States and Brazil [67, 93].

Extracts obtained from annatto seeds and leaves have been tested in cell cultures and the brine shrimp assay, respectively, and have been found to lack cytotoxicity in either model used. These experiments were carried out in Guatemala and the Dominican Republic [83, 89].

On the other hand, a study performed in Cuba with 10 medicinal plants that were active in inhibiting human lung carcinoma cell growth showed that the ethanolic extract of* Bixa orellana* presented cytotoxicity at concentrations below 100 *μ*g/mL [81].

## 8. Toxicological Activities

Currently, concerns about the effect of synthetic dyes on human health are incontestable, making people increasingly choose those of natural origin, believing that they are devoid of toxic effects. This is not entirely true, because even a medication from a natural source can be a poison, depending on the dose that is administered. The failure to require in-depth data related to toxicological and chemical analyses for the registration of food additives derived from natural sources [67, 74] certainly makes the information about possible unwanted effects and/or pharmacological activities resulting from their use, much rarer than expected in view of the importance of the topic. In Brazil, the use of annatto is so widespread that its safety is not even questioned.

Paumgartten et al. [91] evaluated the toxicity of annatto extracts in rats. Doses up to 500 mg/kg body weight/day were introduced directly into the stomach of pregnant rats to evaluate the effect on the mother and fetus, and no adverse effects were found for either. The annatto extract did not induce an increase in the incidence of visible external, visceral, or skeletal anomalies in the fetuses. Therefore, the study suggested that the annatto extract was not toxic to rats nor was it embryotoxic. Studies performed in Brazil by Alves de Lima et al. [67], where extracts of annatto were mixed with the food of male rats, showed that the concentrations tested had no mutagenic or antimutagenic activity in their bone marrow cells. A parallel toxicity study conducted by Hagiwara et al. [74] showed that 0.1% annatto extract administered for thirteen weeks in the feed of male and female rats did not show any adverse effects. However, when higher doses were administered (0.3 and 0.9%), the authors noticed an increase in liver weight as well as changes in blood chemistries, including increase in alkaline phosphatase, phospholipids, and total protein, as well as albumin and albumin/globulin ratio.

Hagiwara et al. [74] also evaluated extracts of annatto for liver carcinogenicity in rats and found no evidence of liver tumors, even when given to animals at a high dose of 200 mg/kg body weight/day, compared to an acceptable dose of 0.0 to 0.065 mg/kg/day, thus indicating that the danger of a hepatocarcinogenic effect in humans may be absent or negligible.

A toxicity test was performed with extracts obtained from both plant seeds and shoots, and no significant effect was observed. The experiments were performed in the United States using mice as the animal model and it was found that the LD50 was greater than 700 mg/kg [92].

## 9. Correlation between the Biological Activities, Phytochemistry, and the Traditional Uses of* Bixa orellana*

Table 1 shows that many of the traditional uses of* Bixa orellana* are the same in several countries of South and Central America, which suggests its effectiveness as a therapeutic agent. Extracts of* Bixa orellana* showed biological activities such as antioxidant, hypotensive, molluscicide, and antimalarial against A549 cells for carcinoma of the lung, allergy, hypoglycemic, antifungal, antioxidant, insect repellent, antigonococcal, and antivenom serum and some of them are in accordance with the traditional use; for example, in Brazil it is used to extract the seeds with purpose repellent insecticide and antimalaria and scientific studies in the same country with the* Lutzomyia longipalpis* insect repellent action and prove a study in Cuba proved the pharmacological action for antimalarial activity when tested against* Plasmodium berghei*. Some of them are in accordance with the traditional use; for example, seed extracts have been used in Brazil and Cuba as insect repellent and antimalarial. Antioxidant and insect repellent activities can be attributed to the carotenoids and the essential and fixed oils, respectively.

Despite the previous reports about the presence of components with anti-inflammatory properties, such as salicylic acid, lutein, polyphenols, and tannins, this activity has not yet been proven for* Bixa orellana* extracts. Similarly, the plant's essential and fixed oils have shown antibacterial properties, although this activity has not been proven too.

In general, the data obtained in this review do not allow correlations between the biologic activities tested* in vitro* or* in vivo* with the compounds identified in this species. However, taking into account the related activities such as the antiparasitic effect and the lack of mutagenic and cytotoxicity activity, it is possible to consider* Bixa orellana* as a potential source for the development of phytopharmaceutical products.

In conclusion, the studies discussed in this review represent a rich database around the* Bixa orellana* activities and its potential uses, which evokes the feasibility of phytopharmaceuticals to treat some diseases whenever an antioxidant, hypotensor, or hypoglycemiant activity is necessary.

Although the commercial exploitation of this species is well established, there are very few studies on its pharmacological effects. Considering the need for developing a safe and effective product, more studies should be performed in order to confirm other biological activities supported by the popular uses of* Bixa orellana.*

## Figures and Tables

**Table 1:** Traditional uses of annatto in American countries.

Country/use	Plant part	References
Argentina		
Antipyretic/cardiotonic/antidiarrheal	Seeds	[24]
Antidiarrheal/dyes/condiment	Seeds	[25]
Brazil		
Body paint	Seeds	[26]
Insect repellent	Seeds	[27]
Condiment/food coloring	Seeds	[27]
Antipyretic	Seeds	[26]
Antipyretic/laxatives/burns	Seeds	[28]
Malaria	Seeds	[26]
Colombia		
Snakebite	Leaves	[29]
Aphrodisiac	Seeds	[30]
Cuba		
Aphrodisiac	Seeds	[31]
Guatemala		
Gonorrhea/dysentery	Leaves	[32]
Hepatitis	Leaves	[33]
Dysentery	Leaves	[34]
Blood diseases	Leaves	[35]
Gonorrhea	Roots	[32]
Diabetes	Roots	[36]
Honduras		
Aromatic/food coloring	Seeds	[37]
Pain/digestive/dysentery	Leaves	[38]
Jamaica		
Diabetes	Seeds	[39]
Nicaragua		
Respiratory and pulmonary disorders/diarrhea/diuretic/burns	Leaves + seeds	[40]
Labor pains	Seeds	[41]
Cough/cold/diuretic/diarrhea/burns/labor pains	Seeds	[40]
Paraguay		
Insecticide/repellent	Seeds	[42]
Diabetes	Seeds	[39]
Peru		
Aphrodisiac/aphrodisiac/diuretic/antidisenteria/astringent	Fruits	[34]
Antipyretic/skin problems	Leaves	[33]
Alcoholic hepatitis/worms	Roots	[33]
Antipyretic/aphrodisiac/dysentery/astringent/stomach	Seeds	[34]
Trinidad and Tobago		
Diuretic	Leaves	[43]
Diabetes	Roots	[44]
Diabetes	Roots	[45]

**Table 2:** The main carotenoids from the seeds of *Bixa orellana*.

Carotenoid	Country of isolation	Physical aspect	References
Apo-*ψ*-carotene, 9′*Z*-6′-ol	Brazil	Oil	[46]
Beta carotene	Brazil	183°C	[47]
	Suriname	∗	[48]
Bixin	Brazil	198°C	[49–51]
	Peru	∗	[52]
	Dominican Rep.	∗	[53]
	Suriname	∗	[48]
	USA	∗	[54]
*Z*-Carotene	Brazil	Oil	[46]
Cryptoxanthin	Suriname	173°C	[48]
Dimethyl-(9*Z*)-6,6′-diapocarotene-6,6′-dioate	Brazil	Oil	[52]
Dimethyl-(9*Z*,9′*Z*)-6,6′-diapocarotene-6,6′-dioate	Brazil	Oil	[50]
Phytoene	Brazil	Oil	[46]
Phytofluene	Brazil	Oil	[46]
Geranylgeraniol	Brazil	Oil	[52]
Lutein	Suriname	196°C	[48]
Methyl-(9*Z*)-10′-oxo-6,10′-diapocarotene-6-oate	Brazil	Oil	[50]
Methyl-(9*Z*)-6′-oxo-6,5′-diapocarotene-6-oate	Brazil	Oil	[50]
Methyl-(9*Z*)-8′-oxo-6,8′-diapocarotene-6-oate	Brazil	Oil	[50]
Methyl-(9′*Z*)-*apo*-6′-lycopenoate	Brazil	Oil	[46]
Methyl-(7*Z*,9*Z*,9′*Z*)-*apo*-6′-lycopenoate	Brazil	Oil	[55]
Methyl-(9*Z*)-*apo*-8′-lycopenoate	Brazil	Oil	[46]
Methyl-(all-*E*)-*apo*-8′-lycopenoate	Brazil	Oil	[46]
Neurosporene	Brazil	Oil	[46]
Norbixin	Brazil	300°C	[52]
*Trans*-bixin	Jamaica	195°C	[56]
Zeaxanthin	Suriname	215°C	[48]

Source: [57].

**Table 3:** Biological activities of extracts of annatto in American countries.

Country biological activity	Part used	Type of extract	Organism tested	Model tested	Dose used	Activity	References
Argentina							
Antibacterial	LE	EtOH	*Bacillus subtilis *	*In vitro *	5 mg/mL	Inactive	[70]
			*Escherichia coli *	*In vitro *	5 mg/mL	Inactive	[70]
			*Micrococcus luteus *	*In vitro *	5 mg/mL	Inactive	[70]
			*Pseudomonas aeruginosa *	*In vitro *	5 mg/mL	Inactive	[70]
			*Staphylococcus aureus *	*In vitro *	5 mg/mL	Inactive	[70]
Antifungal	LE	EtOH	*Aspergillus niger *	*In vitro *	5 mg/mL	Inactive	[70]
			*Candida albicans *	*In vitro *	5 mg/mL	Inactive	[70]
Antiviral	SE	EtOH	Virus *Herpes simplex 1 *	Cell culture	0.78 mg/mL	Inactive	[71]
Insecticidal	AP	MeOH	Insect	*Sitophilus oryzae *	5%	Inactive	[71]
Brazil							
Antimalarial	SE	CHCl_3_	Mouse	*Plasmodium berghei *	100 mg/kg	Inactive	[26]
Antioxidant	SE	EtOH	*In vitro *	DPPH assay	0.1 g L^−1^	Active	[6]
Insect repellent	SE	Petr. eth	Hamster	*Lutzomyia longipalpis *	1 g/L	Active	[72]
	SE	EtOH	Mosquito	*Aedes aegypti *	18.2 mg/mL	Active	[6]
Molluscicidal	SE	EtOH	Conch	*Biomphalaria glabrata *	10,000 ppm	Inactive	[73]
Mutagenic		Powder	Mouse	Bone marrow cells	10,670 ppm	Inactive	[67]
Toxicity	SE	Powder	Rat	*In vivo *	500 mg/kg	Inactive	[74]
Antileishmanial	LE/RO	EtOH	*Leishmania amazonensis *	*In vitro *	0,12–2,5 mg/mL	Active	[75]
Antileishmanial	SE	OE	*Leishmania amazonensis *	*In vitro *	10/50/100/500/1000 *μ*g/mL	Active	[76]
Hyperlipidemia	SE	H_2_O	Mouse	*In vitro *	400 and 800 mg/kg	Active	[77]
Colombia							
Snakebite	LE	EtOH	Mouse	*Bothrops atrox *	LD50 > 260 *μ*g/animal	Active	[29]
Costa Rica							
Anti-inflammatory	RO	EtOH	Rat	Paw edema/carrageenan	100 mg/kg	Inactive	[78]
Cuba							
Positive inotropic effect	AP	H_2_O	Guinea pig	Isolated atrium	320 *μ*L	Inactive	[79]
Antimalarial	SE		*Plasmodium gallinaceum/falciparum *	*In vivo/in vitro *	500 mg/kg	Active	[80]
Cytotoxic	SE	EtOH	Tumor cells	*In vitro *	3,9–250 mg/mL	Active	[81]
Ecuador							
Antifungal	LE	MeOH	*Aspergillus niger *	*In vitro *	10 mg/disk	Inactive	[82]
			*Candida albicans *	*In vitro *	10 mg/disk	Inactive	[82]
			*Cryptococcus neoformans *	*In vitro *	10 mg/disk	Inactive	[82]
			*Fusarium oxysporum *	*In vitro *	10 mg/disk	Inactive	[82]
			*Neurospora crassa *	*In vitro *	10 mg/disk	Inactive	[82]
			*Penicillium purpurogenum *	*In vitro *	10 mg/disk	Inactive	[82]
			*Trichophyton mentagrophytes *	*In vitro *	10 mg/disk	Active	[82]
Guatemala							
Antibacterial	LE	Various	*Escherichia coli *	*In vitro *	50 *μ*L/disk	Inactive	[83]
			*Pseudomonas aeruginosa *	*In vitro *	MIC > 10 mg/mL	Inactive	[83]
			*Salmonella typhi *	*In vitro *	MIC > 10 mg/mL	Inactive	[83]
			*Shigella dysenteriae *	*In vitro *	50 *μ*L	Inactive	[83]
			*Staphylococcus aureus *	*In vitro *	MIC > 10 mg/mL	Inactive	[83]
Antifungal	LE	H_2_O	*Aspergillus flavus *	*In vitro *	MIC > 10 mg/mL	Inactive	[84]
			*Candida albicans *	*In vitro *	MIC > 10 mg/mL	Inactive	[84]
			*Microsporum gypseum *	*In vitro *	MIC > 10 mg/mL	Inactive	[84]
Antigonorrheal	LE	EtOH	*Neisseria gonorrhea *	*In vitro *	50 *μ*L/disk	Active	[32]
Antitrypanosomal	LE	EtOH	*Trypanosoma cruzi *	*In vitro *	MIC > 1 mg/mL	Inactive	[83]
Cytotoxic	LE	H_2_O	Crustacean	*Artemia salina *	LC50 > 1,000 ppm	Inactive	[83]
Inhib. of platelet aggregation	SE	/	*In vitro *	Thrombin aggregation	IC50 0.795 mg/mL	Inactive	[35]
Hawaii							
Contraceptive	RO	/	Mouse	/	0.2 mL/animal	Inactive	[85]
Jamaica							
Hypoglycemic	SE	CHCl_3_	Dog		1 g	Active	[39]
		H_2_O	Dog		200 mL/animal	Active	[39]
Antioxidant	SE	EtOH	*In vitro *	*In vitro *	0.25 and 2.5 *μ*g/mL	Active	[86]
Mexico							
Allergenic	SE	Oil	Human		25 *μ*L/ person	Active	[87]
Paraguay							
Insecticidal	SE	Petr. ether	Insect	*Rhodnius neglectus *	50 *μ*g	Inactive	[42]
Puerto Rico							
Molluscicidal	TP	H_2_O	Conch	*Lymnaea cubensis *	LD100 > 1 M ppm	Inactive	[88]
Dominican Republic							
Cytotoxic	SE	EtOH	Cell culture	Molt 4 cells	200 *μ*g/mL	Inactive	[89]
Trinidad and Tobago							
Antibacterial	SE	EtOAc	*Escherichia coli *	*In vitro *	1,000 *μ*g/mL	Inactive	[90]
			*Pseudomonas aeruginosa *	*In vitro *	1,000 *μ*g/mL	Inactive	[90]
USA							
Anticonvulsant	RA	EtOH	Mouse	Seizures/electroshock	100 mg/kg	Inactive	[91]
			Rat	Seizures/pentylenetetrazole	400 mg/kg	Inactive	[91]
Antimalarial	SE	CHCl_3_	Chicken	*Plasmodium gallinaceum *	388 mg/kg	Inactive	[92]
Mutagenic	SE	MeOH	*Salmonella typhimurium *		100 mg/plaque	Inactive	[93]
		H_2_O	*In vitro *	Placental trophoblasts	100 mg/plaque	Inactive	[93]
Hypotensive	RA	EtOH	Rat		50 mg/kg	Active	[93]
Toxicity	RA	H_2_O	Mouse		LD50 > 700 mg/kg	Inactive	[92]

LE: leaf; AP: aerial part; TP: total plant; RO: root; SE: seed; /: not given; LD50: 50% lethal dose; IC50: concentration that inhibits 50% of the effect; MIC: minimum inhibitory concentration.
